# Longitudinal Microbiome Composition and Stability Correlate with Increased Weight and Length of Very-Low-Birth-Weight Infants

**DOI:** 10.1128/mSystems.00229-18

**Published:** 2019-02-26

**Authors:** Alyson L. Yee, Elizabeth Miller, Larry J. Dishaw, Jessica M. Gordon, Ming Ji, Samia Dutra, Thao T. B. Ho, Jack A. Gilbert, Maureen Groer

**Affiliations:** a Interdisciplinary Scientist Training Program, University of Chicago, Chicago, Illinois, USA; b Microbiome Center, University of Chicago, Chicago, Illinois, USA; c Department of Pediatrics, Morsani College of Medicine, University of South Florida, Tampa, Florida, USA; d Department of Surgery, University of Chicago, Chicago, Illinois, USA; e Argonne National Laboratory, Chicago, Illinois, USA; f College of Nursing, University of South Florida, Tampa, Florida, USA; g Department of Anthropology, College of Arts and Sciences, University of South Florida, Tampa, Florida, USA; University of California, Irvine

**Keywords:** VLBW infant, infant growth, infant microbiome, longitudinal microbiome, preterm birth

## Abstract

The microbiomes of 83 preterm very-low-birth-weight (VLBW) infants and clinical covariates were analyzed weekly over the course of their initial neonatal intensive care unit (NICU) stay, with infant growth as the primary clinical outcome. Birth weight significantly correlated with increased rate of weight gain in the first 6 weeks of life, while no significant relationship was observed between rate of weight gain and feeding type. Microbial diversity increased with age and was significantly correlated with weight gain and percentage of the mother’s own milk. As expected, infants who received antibiotics during their NICU stay had significantly lower alpha diversity than those who did not. Of those in the cohort, 25 were followed into childhood. Alpha diversity significantly increased between NICU discharge and age 2 years and between age 2 years and age 4 years, but the microbial alpha diversity of 4-year-old children was not significantly different from that of mothers. Infants who showed improved length over the course of their NICU stay had significantly more volatile microbial beta diversity results than and a significantly decreased microbial maturity index compared with infants who did not; interestingly, all infants who showed improved length during the NICU stay were delivered by Caesarean section. Microbial beta diversity results were significantly different between the time of the NICU stay and all other time points (for children who were 2 or 4 years old and mothers when their children were 2 or 4 years old).

**IMPORTANCE** Preterm infants are at greater risk of microbial insult than full-term infants, including reduced exposure to maternal vaginal and enteric microbes, higher rates of formula feeding, invasive procedures, and administration of antibiotics and medications that alter gastrointestinal pH. This investigation of the VLBW infant microbiome over the course of the neonatal intensive care unit (NICU) stay, and at ages 2 and 4 years, showed that the only clinical variables associated with significant differences in taxon abundance were weight gain during NICU stay (*Klebsiella* and *Staphylococcus*) and antibiotic administration (*Streptococcus* and *Bifidobacterium*). At 2 and 4 years of age, the microbiota of these VLBW infants became similar to the mothers’ microbiota. The number of microbial taxa shared between the infant or toddler and the mother varied, with least the overlap between infants and mothers. Overall, there was a significant association between the diversity and structure of the microbial community and infant weight and length gain in an at-risk childhood population.

**Author Video**: An author video summary of this article is available.

## Abstract (precis)

Preterm infants are at greater risk of microbial insult than full-term infants, including reduced exposure to maternal vaginal and enteric microbes, higher rates of formula feeding, invasive procedures, and administration of antibiotics and medications that alter gastrointestinal pH. This investigation of the VLBW infant microbiome over the course of the neonatal intensive care unit (NICU) stay, and at ages 2 and 4 years, showed that the only clinical variables associated with significant differences in taxon abundance were weight gain during NICU stay (*Klebsiella* and *Staphylococcus*) and antibiotic administration (*Streptococcus* and *Bifidobacterium*). At 2 and 4 years of age, the microbiota of these VLBW infants became similar to the mothers’ microbiota. The number of microbial taxa shared between the infant or toddler and the mother varied, with least the overlap between infants and mothers. Overall, there was a significant association between the diversity and structure of the microbial community and infant weight and length gain in an at-risk childhood population.

## INTRODUCTION

Humans have canonically been considered sterile *in utero*, with the majority of microbial colonization occurring during and immediately following birth. This early microbial exposure begins a dynamic process, activating the immature immune system and leading to the selection of particular microbes. At birth, gut-associated lymphoid tissue is “uneducated” and the innate immune response to the presence of bacteria is crucial for the transition from fetal to postnatal life (1, 2). Recent evidence suggests that there are time-dependent windows of development that are contingent upon microbial signals to trigger maturation of mucosal immunity. For example, the microbiome at 2 months of age has been shown to be predictive of a child’s interleukin expression levels at 2 years of age (3). Indeed, children with altered microbiome development show an increased incidence of immune-mediated disorders in adulthood (4–7). This implies that perturbations or abnormal assembly during this early life interval may have lifelong health consequences.

The ecological succession of the human microbiome in healthy, full-term infants has been well documented and shows a patterned progression from birth to an “adult-like” state around 2.5 years postpartum (8, 9). Initial colonizers of the infant gut are typically facultative anaerobes, shifting to obligate anaerobes within days or weeks (10) as the maturing colonocytes start to scrub oxygen from the intestinal lumen (11). In preterm infants, the microbiome undergoes a similar pattern of development, but does so after a significant delay (12). The preterm infant microbiome is characterized by low diversity and low stability, as well as by an abundance of opportunistic pathogens (13). Microbiota development in premature infants appear to be associated with gestational age but is also shaped by maternal environment and lifestyle and by the unique environment and clinical practices in the neonatal intensive care unit (NICU) (14–17). Preterm infants are exposed to high levels of antibiotics and low levels of maternally derived microbes, leaving them particularly susceptible to colonization from an environmental source, such as the NICU built environment, which may harbor antibiotic resistance genes (13, 18). Furthermore, a recent study of late-preterm infants, who are likely to receive postnatal care similar to that received by full-term infants, determined that late-preterm birth independently affected gut microbiome development during the first 6 months of life (19).

The goals of this study were to analyze the gut microbiome during the first 6 weeks after birth for very-low-birth-weight (VLBW) infants and to determine the associations between the variance in microbial profile and infant health outcomes. Subsequently, 25 of the original 83 children were reexamined at ages 2 and 4, and the stool microbial profile was assessed with respect to health, development, and growth (weight and length) outcomes. We hypothesized that microbial community structure present in the first 6 weeks of the NICU stay is statistically associated with mode of delivery, gestational age, weight and length gain, type of feeding, human milk cytokines, fecal calprotectin, and adverse prenatal events and that the microbial community structure of the infant gut microbiome can be used to predict the microbial community structure in the same child at 2 and 4 years of age.

## RESULTS

### Overview of the preterm infant cohort.

Preterm infant clinical variables (see Table S1 in the supplemental material) were determined for a population cohort of 83 preterm infants delivered at Tampa General Hospital between May 2012 and Dec 2013. Each child was sampled for fecal matter approximately weekly for up to 6 weeks during the NICU stay and was then followed up at 2 years old and 4 years old. Of the 83 subjects in the original sample, only about 57 were locatable at 2 years of age, and 25 were recruited from that group for follow-up, which significantly impacted the statistical power of our analyses to develop a predictive model of the microbial community structure between the NICU stay and childhood. The longitudinal analysis comprised 425 fecal samples, which were processed for 16S rRNA amplicon sequencing to characterize the microbiome.

10.1128/mSystems.00229-18.3 TABLE S1 Patient characteristics. Data represent maternal demographics and interventions during NICU stay. Download Table S1, DOCX file, 0.1 MB. Copyright © 2019 Yee et al. 2019 Yee et al. This content is distributed under the terms of the Creative Commons Attribution 4.0 International license.

Relevant available age metrics included gestational age at birth, chronological age, and postmenstrual age. Infants in our cohort had a wide range of gestational ages at birth (24 to 37 weeks). Therefore, chronological age was not employed as the independent variable; instead, we used postmenstrual age (chronological age plus gestational age; also known as corrected gestational age) for all subsequent analyses. A total of 91.6% of the infants started antibiotics on first day of life (regardless of gestational age). The mean postmenstrual age for starting antibiotics was 28.2 ± 2.5 (standard deviation [SD]) weeks. Most infants were on antibiotics for 3 to 4 days at a time, with a median course of antibiotics lasting 3.5 days. Only 7 infants did not receive any antibiotics. The mean total number of days on antibiotics was 11.88 ± 18.8 (range, 0 to 114 days).

In total, 6,383,544 16S rRNA amplicon reads were generated across all 425 samples, with a range of ∼1,000 to 40,483 reads (median, 13,092; mean, 15,020.1). Sequence depth was rarified to 1,275 reads per sample, resulting in 415 samples used in subsequent analysis. Reads were classified into exact sequence variants (ESVs) using DADA2, creating a total of 2213 ESVs, with a range of 1 to 1,844,477 reads per sample (median, 48; mean, 2,884.5). All ESVs were annotated to known microbial taxa.

### Infant growth as a clinical outcome.

Our analyses were statistically underpowered for each morbidity category (only 5 infants with chronic lung disease [CLD], 3 with necrotizing enterocolitis [NEC], 10 with blood culture-positive sepsis, and 9 with any stage of intraventricular hemorrhage [IVHn]); therefore, we focused on growth (increased weight and length) as a primary clinical outcome during the NICU stay. We tested the independence of the measured growth variables (Materials and Methods). Birth weight (in grams) was significantly correlated with growth rate over the first 6 weeks of life (grams/week) (see Fig. S1 in the supplemental material). Overall, there was a weak but significant association between higher initial birth weight and faster initial weight gain (*R*^2^ = 0.146, *P* value = 0.001703); however, this relationship was not significant for birth weight and weight gain between 6 weeks and discharge (*P* = 0.8141). No significant relationships were observed between weight at discharge and weight gain with feeding (total volume of milk received and ratio of mother’s breast milk volume to total enteral feeding volume received; *P* > 0.2).

10.1128/mSystems.00229-18.1 FIG S1 Birth weight (in grams) correlates with growth per week initially (first 6 weeks). *R*^2^, 0.146; *P* value, 0.001687. Download FIG S1, PDF file, 0.1 MB. Copyright © 2019 Yee et al. 2019 Yee et al. This content is distributed under the terms of the Creative Commons Attribution 4.0 International license.

### Microbial diversity increased with age and was statistically associated with clinical variables.

**FIG 1:**
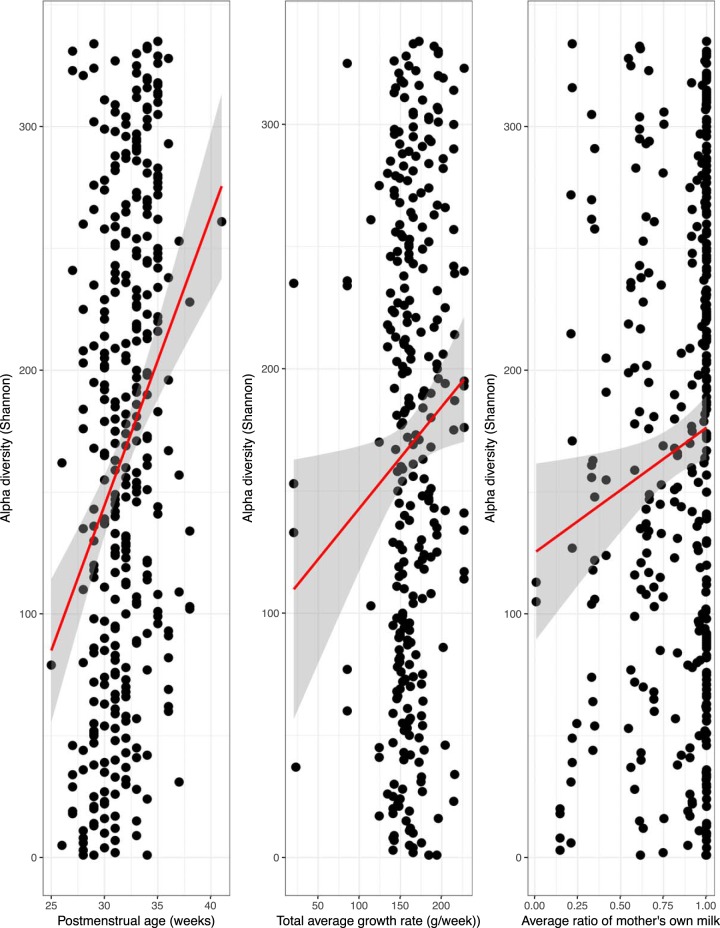
Correlation of alpha diversity with (left panel) postmenstrual age (Shannon: multiple *R*^2^ value, 0.1508; *P* value, 1.507e−06), (middle panel) total growth rate (in grams/week), and (right panel) ratio of the mother’s own milk volume to total milk volume.

The relationship between weight and length gain outcomes and microbial alpha diversity over the course of the NICU stay was determined. Shannon index values increased significantly with increasing postmenstrual age (multiple *R*^2^, 0.09221; *P* = 1.28e−08) (Fig. 1, left panel). Adjusting for postmenstrual age during the NICU stay, the alpha diversity also showed weak but significant correlations with the overall rate of weight gain (multiple *R*^2^, 0.144; *P* = 0.00208) (Fig. 1, middle panel) and the total percentage of the mother’s own milk (MOM; multiple *R*^2^, 0.1097; *P* = 0.024918) (Fig. 1, right panel), but no statistically significant associations were observed with the following other clinical variables: mode of delivery, length of hospitalization in the NICU, time to full enteral feeding, days on antibiotics, infant weight at birth or discharge, days on oxygen, APGAR (appearance, pulse, grimace, activity, and respiration) scores at 1 or 5 min postbirth, duration of breast-feeding, or maternal age. We found no significant associations with microbial alpha diversity and common comorbidities, although our analyses lacked statistical power within the cohort for each morbidity. As expected, infants who received antibiotics during the NICU stay had significantly lower alpha diversity than those who did not receive antibiotics (*t* test, false-discovery-rate [FDR] corrected, *P* = 0.009). Values for Shannon alpha diversity difference per infant between the first NICU stool sample and the last stool sample ranged from −3.3520 to 3.1720, with a median of 0.8259. There were no significant differences with respect to delta Shannon alpha diversity between children who showed improved length and those who did not or by mode of delivery (*P* > 0.3; *t* test, FDR correction).

A significant increase in Shannon index values was also observed for each child in comparisons between their last time point of NICU stay and 2 and 4 years of age (*P* = 0.01; *t* test); the Shannon index values were also significantly different in comparisons of 2 and 4 years of age. While the alpha diversity during the NICU stay and at 2 years of age was significantly lower than the alpha diversity of the mother’s stool-associated microbiota (*P* = 0.01; *t* test), the alpha diversity at 4 years of age was not significantly different from that of the mother, suggesting that the diversity normalizes to that of an adult by 4 years of age.

### Microbial beta diversity, maturity, and microbial stability.

The distribution of between-sample unweighted UniFrac distances was calculated pairwise to determine the volatility of microbial community structure over time during the NICU stay. This was then associated with the clinical variables, including both categorical variables (delivery method, sex, gestational age, weight for gestational age, improved length, multiples, weight gain rate, ratio of volume of breastmilk to total volume of milk received, length of stay in NICU) and binary variables (antibiotic treatment, sepsis, NEC, CLD, transfusions, feeding intolerance). Beta diversity volatility was significantly correlated with improved length only during the NICU stay, with results indicating that infants who showed improved length had significantly more intersample variability in beta diversity and hence had greater microbial volatility over the course of their NICU stay (*P = *0.02805; FDR corrected). This suggests that infants with a less-volatile microbiota over time may have reduced catch-up growth (length) during the NICU stay. While the mode of delivery alone did not correlate with volatility, all the infants with improved length (*n* = 6) had been delivered by Caesarean section.

**FIG 2:**
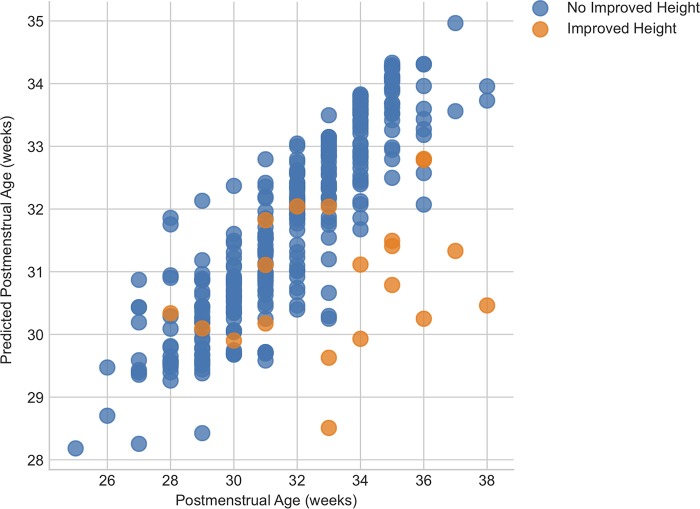
Maturity analysis data showing actual versus predicted gestational age at birth for infants showing or not showing improved length.

We ran a “maturity” analysis (Materials and Methods), which uses a regression model to predict postmenstrual age as a function of the composition of the microbiota. Therefore, if a sample had a microbiota which fell outside the confidence interval based on the average microbiota composition for infants of each postmenstrual age, it was considered immature. We correlated the maturity index with improved length during their NICU stay (Fig. 2). Those infants who did have improved length during their NICU stay had a significantly reduced level of predicted microbial maturity compared to infants whose length did not significantly increase (*P* < 0.0018). As maturity is inversely correlated with beta diversity volatility, this result agrees with the results of the beta diversity volatility analysis described above.

**FIG 3:**
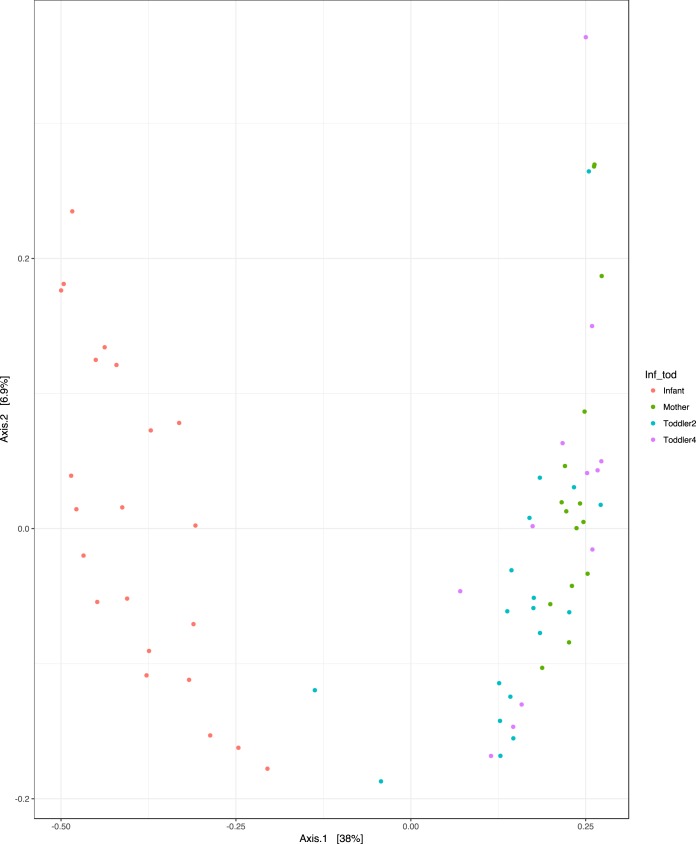
Unweighted Unifrac Beta diversity PCoA of infant samples (i.e., those from the last NICU time point) and samples from 2-year-old children, 4-year-old children, and mothers.

We also examined the beta diversity distribution (unweighted UniFrac) to determine the microbial community structure variance between the final NICU time point, age 2 years and age 4 years, and maternal samples. The maternal microbiota and those of the 2-year-old and 4-year-old children also clustered together and were significantly different from those seen with the samples collected at the end of the NICU stay (Fig. 3; principal-coordinate analysis [PCoA], *P < *0.05 after FDR).

### Microorganisms associated with clinical outcomes and age.

The stool samples collected during the NICU stay maintained a predominance of *Gammaproteobacteria*, specifically, *Enterobacteriaceae*, members of which are often associated with lipopolysaccharide (LPS)-mediated inflammation. The most abundant exact sequence variant (ESV) in infants during the NICU stay was closely related to the genus *Klebsiella*, which belongs to the *Enterobacteriaceae*. Analysis of composition of microbiomes (ANCOM) was applied against all categorical and binary clinical variables to determine which bacteria were significantly differentiated in relative abundance. Only two clinical variables showed bacteria that were significantly differentially abundant. Infant weight gain from birth to discharge was significantly negatively correlated with the relative abundance of two ESVs, one associated with *Klebsiella* and the other related to *Staphylococcus* (*P* < 0.01). Additionally, antibiotic administration was associated with significant enrichment in *Proteus* levels and a significant proportional decrease in the levels of *Streptococcus* and *Bifidobacterium* compared to infants who did not receive antibiotics (*P* < 0.01). The predominance of *Proteobacteria* was greater in infants with lower beta diversity volatility than in those with higher volatility.

10.1128/mSystems.00229-18.2 FIG S2 Relative abundances of taxa at the family level. Each bar represents a single sample. Bars are sorted by patient identifier (ID) and ordered by age for each patient. Download FIG S2, PDF file, 0.3 MB. Copyright © 2019 Yee et al. 2019 Yee et al. This content is distributed under the terms of the Creative Commons Attribution 4.0 International license.

The random forest classifier was used to determine whether the microbiota was predictive of categorical clinical variables (clinical factors that could be grouped into discrete components), with results which suggested that only the use of antibiotics (presence/absence) could be accurately predicted, with a baseline-to-random ratio of >2 (2.26) suggesting a marginal relationship. The random forest features identified as having the most predictive value for the presence or absence of antibiotics were ESVs corresponding to *Enterobacteriaceae*, *Citrobacter*, *Escherichia*, and *Peptostreptococcaceae*. For continuous clinical variables, we also ran the random forest regressor model (1,000 estimators, bootstrapping, mean square error [MSE]), which identified only the duration of antibiotic administration as significantly predictable (*R*^2^ of 0.16259; *P = *0.000651). The top most important features for the regressor-model accuracy were ESVs corresponding to *Veillonella*, *Enterobacteriaceae*, *Proteus*, and *Escherichia* (importance scores of 0.15 to 0.05).

**FIG 4:**
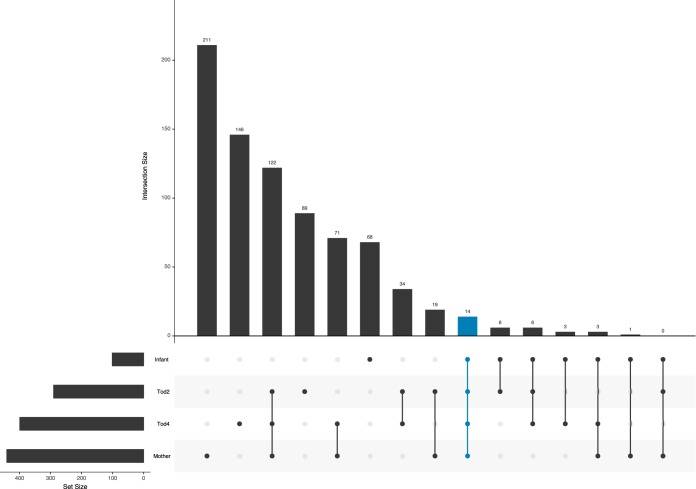
UpSet plot showing ESVs shared between infant samples and samples from 2-year-old children, 4-year-old children, and mothers. The numbers of unique ESVs shared between groups or intersections of groups are plotted as vertical bars. The 14 “core” ESVs shared between all four sample groups are indicated in blue.

While the infant samples at 6 weeks of life had a significantly greater abundance of ESVs related to *Enterococcus* and *Staphylococcus* (Kruskal-Wallace; Bonferroni-corrected *P* < 0.0001), samples from toddler at both 2 and 4 years old maintained ESVs associated with *Clostridiales* and *Bacteroidales* that were not detected in the infant samples for the respective individuals. However, the infants as a group shared 26 ESVs in total with 2-year-old toddlers as a group, 26 ESVs in common with 4-year-old toddlers, and 18 ESVs in common with the mothers as group (Fig. 4). Interestingly, the 2-year-old toddlers shared 155 ESVs with their mothers, while the 4-year-old children shared 210 ESVs with their mothers. This suggests that the number of ESVs shared with the mother increases with increasing age, likely due to the changing gut environment and an increase in food similarity. In total, only 14 ESVs were shared between infants, 2-year-old and 4-year-old children, and mothers; those comprise the core ESVs. The ESVs included genera such as *Ruminococcus*, *Oscillospira*, *Bacteroides*, *Streptococcus*, *Bifidobacterium*, *Escherichia*, *Eggerthella*, and *Dorea* and families such as *Lachnospiraceae*, *Peptostreptococcaceae*, and *Rikenellaceae* (Table S2). Interestingly, one of the core ESVs found in all age groups was related to Haemophilus parainfluenzae, which is a potential pathogen. Overall, the *H. parainfluenzae* ESV was found in ∼10% of the samples, from 29% (24) of the participants. Some infants maintained the H. parainfluenzae ESV over multiple NICU time points, but it was not found in the corresponding 2-year-old and 4-year-old samples for those infants. However, it was found in 3 children at both 2 years of age and 4 years of age and also in their mothers. As mothers’ stool samples were collected at the same time as the stool samples collected from the 4-year-olds, it is possible that this H. parainfluenzae ESV represented endemicity in these 3 families during that time.

10.1128/mSystems.00229-18.4 TABLE S2 Taxonomy of ESVs in common between all age groups (last NICU time point, 2 years, 4 years, and age of mother in years). Download Table S2, DOCX file, 0.1 MB. Copyright © 2019 Yee et al. 2019 Yee et al. This content is distributed under the terms of the Creative Commons Attribution 4.0 International license.

## DISCUSSION

Preterm infants are particularly vulnerable to perturbations in gut microbiome development due to their abnormal delivery and prolonged NICU stay, which is associated with many insults. Our study results complement prior observations showing that the preterm infant microbiome is characterized by an abundance of *Proteobacteria* (20, 21). Bacterial alpha diversity was significantly positively correlated with postmenstrual age, and, adjusted for postmenstrual age during the NICU stay, the alpha diversity also showed weak but significant correlations with overall rate of weight gain and total volume of the mother’s own milk.

While we did not identify significant differences between infants’ microbial beta diversity data by mode of delivery or feeding type, we did, as expected, find an effect of antibiotic administration, which correlated with a significant enrichment of *Proteus*. *Proteus*, a gammaproteobacterial genus, can be an opportunistic pathogen in humans and is frequently found in hospital settings (22, 23). It has been associated with high rates of biofilm formation and antibiotic resistance, which may explain its increased abundance following antibiotic perturbation in our study. Furthermore, postmenstrual age (gestational age plus chronological age, also known as corrected gestational age) accounted for the majority of variance in beta diversity over time. Infant weight gain during the NICU stay was negatively correlated with the relative abundances of *Klebsiella* and *Staphylococcus*. As both of these taxa are associated with known pathogens, it is possible that their enrichment may be indicative of dysbiosis, which in turn appears to be associated with reduced infant weight and length gain, although to the best of our knowledge this specific association has not been demonstrated previously. However, a previous study demonstrated a significant association between bacterial alpha diversity and body weight and gestational age, which were also associated with an increase in the level of staphylococci (24).

We also demonstrated that the z-score corresponding to catch-up growth in length (improved length) was correlated with increased beta diversity volatility (between-sample beta diversity distance) and decreased maturity (predictive power of regressor modeling) during the NICU stay. Catch-up growth is an important consideration for preterm infants: VLBW infants are vulnerable to extrauterine growth restriction, and caloric and protein deficits result in lower growth velocity and are associated with major neonatal morbidities, including chronic lung disease and retinopathy of prematurity (25). Clinically, it is believed that early catch-up growth (at <2 years, and especially in the first 24 weeks of life) is beneficial to preterm-delivered child health outcomes (26, 27). That microbial beta diversity was more volatile for children who demonstrated greater catch-up growth could be related to the overall microbial profile for this community. Infants with lower volatility had significantly greater predominance of *Proteobacteria*; i.e., they had greater week-to-week stability due to the dominance of a group of potential pathogens (e.g., *Klebsiella*). Infants with more volatility had reduced prevalence of these organisms and as such may have had reduced dysbiosis and improved growth. This report is among the first to document a relationship between microbiome and VLBW infant growth. Previous work found that preterm infants with prolonged microbial transitions had smaller changes in weight-for-age z-scores across time (14). However, little is known about the relationship between preterm/VLBW length gain and microbiome development. Poor length gain, as an indicator of long-term nutritional stress, indicates that microbiome development can have a long-term effect on infant phenotype. Researchers have noted that the preterm microbiome has developmental signatures similar to those seen with undernourished term infants (28), suggesting that the therapeutic approaches may be similar for the two groups. More research is needed to establish the relationship between VLBW length and weight gain and microbiome development.

Due to the limited size of this cohort, it is not possible to determine if this trend is absolutely significant, but the data suggest that further studies using larger cohorts would be useful, as would studies involving the humanization of murine models to demonstrate reduced growth as a transferable phenotype. Those infants who demonstrated reduced microbial maturity also showed improved catch-up growth, likely as a result of the choice of population against which these infants were compared. Our maturity analysis is based on the average maturity data determined for a population of VLBW infants who were essentially quite sick, as infants in that category who showed improved catch-up growth would be statistical outliers against this group. Having a healthy control population might provide a more accurate interpretation of the standard maturity analysis data.

In our cohort, we followed the same children to ages 2 and 4 years. Our analyses had insufficient statistical power to identify clinical outcomes that correlated with microbiome composition, but we did find that the these VLBW infants did develop a microbiome comparable to that of their adult mothers by ages 2 to 4 years. As previously demonstrated, the microbiome of children undergoes patterned progression to become more similar to the maternal microbiome as the children increase in age, with an adult-like microbiome established by 4 years (12). In our cohort of VLBW preterm infants, we also observed shifts in the microbiome and increasing similarity to the maternal microbiome with increasing age. Importantly, many of the ESVs found in infants were also found in 2- and 4-year-olds, as well as in the mothers, suggesting conservation of microbial composition between generations, ages, and environments. And yet the level of ESV overlap in infants and mothers was significantly lower than that in mothers and 4-year-old children, suggesting a gradual shift of the microbial community, likely as a result of changes in the ecological drivers that shape the niche structure of the developing gastrointestinal tract. For example, as the gut matures and the immune system becomes educated and the child starts to eat food more similar to that eaten by the adult mother, there is a selective ecological pressure toward a more similar microbial structure (11, 29–33).

We have demonstrated a significant association between the diversity and structure of the microbial community and infant growth in a significantly at-risk childhood population. While the population had inherent variability, which limits the potential to identify associations between the microbiota and clinical outcomes, compelling correlations between microbial structure, volatility, maturity, and composition and infant weight and length increases were determined that suggest potential biomarkers of dysbiosis in this at-risk population.

## MATERIALS and METHODS

The study was approved by both the hospital and the university Institutional Review Board, and parents gave informed consent for the study in the NICU and agreed to be followed up at a later time for further research.

### Patient demographics and clinical information.

A total of 83 preterm, very-low-birth-weight (VLBW) and extremely low-birth-weight (ELBW) infants (gestational age = 28.44 ± 2.39 weeks, birth weight = 1,086.71 ± 218.49 g) were enrolled as soon as possible after admission to the neonatal intensive care unit (NICU) at Tampa General Hospital, an academic level III center with a single-patient-room floor plan, during the period May 2012 to December 2013. Mothers who were drug abusers or HIV positive were excluded, as were infants who had major congenital anomalies or who were moribund. Maternal and neonatal clinical information was obtained from electronic medical records and an investigator-developed demographic form. Long-term morbidity data were extracted from the Vermont Oxford Network database. Data were collected for a total of 6 weeks after entry into the study unless participants were discharged or transferred earlier. Exact volumes of the mother’s own milk (MOM), formula, fortifier, and donor milk (DM) were collected from the electronic medical record.

### Stool sampling, DNA extraction, and amplicon sequencing.

Stool samples were scooped aseptically from a diaper at approximately the end of each week (with some variation), stored in a sterile tube, brought to the laboratory, and frozen at −80°C until processing. DNA was extracted using a MoBio PowerFecal DNA kit (Qiagen, Carlsbad, CA) with modifications based on the Earth Microbiome Project protocols (www.earthmicrobiome.org). The V4 region of 16S rRNA gene was amplified using PCR with modified 515F and 806R primers, followed by amplicon sequencing using a MiSeq platform (Illumina, San Diego, CA) based on existing protocols to generate ∼100,000 250-bp paired-end reads per sample (34). Raw sequence reads are available through the Sequence Read Archive (SRA) (see below).

### Sequencing data processing.

The V4 region 16S rRNA gene amplicon data were analyzed using the DADA2 plugin (35) in QIIME2 (36). Data from 6 separate sequencing runs were imported into QIIME2-2018.2 and demultiplexed with demux emp-paired. The DADA2 plugin was used for quality control, including filtering phiX reads and chimeric sequences. Trimming was performed to trunclen 140 and trim 20, and the resulting feature tables were merged. Taxonomy was assigned against the Greengenes v13.8 database.

### Statistics and machine learning.

We calculated the correlation between the relative abundances of the sequence variants and NICU health outcomes by regression analysis in R. We employed UniFrac distances to examine microbiome structure comparisons between samples to test differences between groups using multivariate statistical methods such as principal-coordinate analysis (PCoA) and permutational multivariate analysis of variance (PERMANOVA). We performed two-sample *t* tests with FDR correction to examine differences between binary variables. Volatility analysis was performed by comparing unweighted UniFrac distances for exact sequence variants (ESVs) between subgroups. To identify the predictive value of subgroups for microbiome community composition, we applied random forest machine learning (after rarefication to 5,000 sequences/sample [1,000 trees]) and Analysis of Composition of Microbiomes (ANCOM) (37). For continuous clinical variables, we ran the random forest regressor model (1,000 estimators, bootstrapping, mean square error [MSE]). Maturity analysis was applied using the sample classifier plugin in QIIME2 (38). UpSet plots were generated using the R package (39).

### Infant growth metrics.

Infant weight and length were measured at birth and weekly until discharge. Raw weight and length measurements were standardized against the 2013 revision of the Fenton preterm growth chart using the actual-age calculator tool (40). This process converts the raw measurement to z-scores based on means and standard deviations of data representing the reference population at each age. This resulted in a weight-for-age and length-for-age z-score at each time point. Standardizing weights and lengths in this manner allows infants of different sexes and gestational ages to be compared as representatives of one population. In addition, because the Fenton method is intended to represent the ideal growth of preterm infants, it can be used to detect changes in growth status as they age. Therefore, a change in z-score over time can be a useful tool to detect growth faltering (a z-score that becomes smaller) and catch-up growth (a z-score that becomes larger).

Growth (weight gain and length-for-age z-score) over the NICU stay was assessed with the following measurements: (i) total average growth rate (difference between birth weight and discharge weight divided by length of stay), growth rate between birth and 6 weeks of life (difference between birth weight and weight at 6 weeks divided by weight at 6 weeks), and growth rate between 6 weeks of life and discharge (difference between weight at 6 weeks and discharge weight divided by length of stay minus weight at 6 weeks); (ii) weight for gestational age (categorical variables: small for gestational age, average for gestational age, or large for gestational age), and (iii) improved length (binary variable corresponding to whether or not the infant had greater-than-expected length-for-age z-scores during the NICU stay). Complete growth metadata were available for only 78 of the total of 83 infants.

### Data availability.

The study was registered in dbGaP under accession number phs001578.v1.p1. Deidentified metadata and raw forward and reverse sequence reads were associated with each sample via SRA. Raw sequence reads are available through the Sequence Read Archive under accession number SRP171050 (BioProject number PRJNA449987).
