# Supplementary material for: Longitudinal Microbiome Composition and Stability Correlate with Increased Weight and Length of Very-Low-Birth-Weight Infants
Source: mSystems. 2019 Feb 26;4(1):e00229-18. doi: 10.1128/mSystems.00229-18 (PMC6392092; doi:10.1128/mSystems.00229-18)
Supplement: TABLE S1 [file mSystems.00229-18-st001.docx]

|  | Overall | Min | Max |
| --- | --- | --- | --- |
| n | 82 |  |  |
| Maternal Age(years) (mean (sd)) | 28.13 (6.89) | 15.2 | 46.1 |
| Days on Antibiotics (mean (sd)) | 11.73 (18.45) | 0 | 114 |
| Paternal Ethnicity (%) | |  |  |
| African American | 34 (43.0) |  |  |
| Asian Pacific Islander | 3 (3.8) |  |  |
| Caucasian | 20 (25.3) |  |  |
| Hispanic Black | 1 (1.3) |  |  |
| Hispanic White | 18 (22.8) |  |  |
| Native American | 1 (1.3) |  |  |
| Other | 2 (2.5) |  |  |
| Maternal Ethnicity (%) | |  |  |
| African American | 34 (42.0) |  |  |
| Asian Pacific Islander | 2 (2.5) |  |  |
| Caucasian | 25 (30.9) |  |  |
| Hispanic Black | 1 (1.2) |  |  |
| Hispanic White | 15 (18.5) |  |  |
| Native American | 1 (1.2) |  |  |
| Other | 3 (3.7) |  |  |
| Parental Education (%) | |  |  |
| College Graduate | 17 (20.7) |  |  |
| Grammar/Elementary School | 4 (4.9) |  |  |
| High School | 47 (57.3) |  |  |
| Middle School | 8 (9.8) |  |  |
| Post Graduate Degree | 6 (7.3) |  |  |
| Marital Status (%) |  |  |  |
| Divorced Separated | 6 (7.4) |  |  |
| Married/Living With Partner | 32 (39.5) |  |  |
| Single | 42 (51.9) |  |  |
| Widowed | 1 (1.2) |  |  |
| Family Income (%) |  |  |  |
| 15,000-24,999 | 12 (16.7) |  |  |
| 40,000-69,999 | 3 (4.2) |  |  |
| 5,000-14,999 | 27 (37.5) |  |  |
| 70000+ | 9 (12.5) |  |  |
| UNDER 4,999 | 20 (27.8) |  |  |
| Unknown | 1 (1.4) |  |  |
| Working (%) |  |  |  |
| Full Time | 17 (21.2) |  |  |
| No | 56 (70.0) |  |  |
| Part Time | 7 (8.8) |  |  |
| Working hours per week (mean (sd)) | 34.80 (18.10) | 0 | 80 |
| Maternal Height (inches) (mean (sd)) | 64.60 (2.80) | 59 | 69 |
| Maternal pre-pregnancy weight (lbs) (mean (sd)) | 167.20 (46.68) | 79 | 303 |
| Number in house (mean (sd)) | 3.15 (1.34) | 1 | 7 |
| Total pregnancies (mean (sd)) | 3.09 (2.46) | 1 | 11 |
| Full term pregnancies (mean (sd)) | 0.66 (0.95) | 0 | 4 |
| Preterm pregnancies (mean (sd)) | 0.43 (0.69) | 0 | 2 |
| Induced Abortions (mean (sd)) | 0.26 (0.64) | 0 | 3 |
| Spontaneous Abortions (mean (sd)) | 0.74 (1.43) | 0 | 7 |
| Ectopic Pregnancies (mean (sd)) | 0.07 (0.26) | 0 | 1 |
| Multiple pregnancies (mean (sd)) | 0.04 (0.19) | 0 | 1 |
| Living children (mean (sd)) | 0.91 (1.15) | 0 | 5 |
| Prenatal care = Yes (%) | 70 (93.3) |  |  |
| Depression = Yes (%) | 10 (12.5) |  |  |
| Depression treatment (%) | |  |  |
| 0 | 2 (20.0) |  |  |
| Medications | 1 (10.0) |  |  |
| Medications and Counseling | 7 (70.0) |  |  |
| Anxiety = Yes (%) | 10 (12.3) |  |  |
| Anxiety treatment (%) | |  |  |
|  | 69 (84.1) |  |  |
| 0 | 3 (3.7) |  |  |
| COUNSELING | 1 (1.2) |  |  |
| MEDICATION | 3 (3.7) |  |  |
| MEDICATION, COUNSELING | 6 (7.3) |  |  |
| Breastfeeding = Yes (%) | 76 (93.8) |  |  |
| Breastfeeding exclusive = Yes (%) | 51 (64.6) |  |  |
| Breastfeeding months (mean (sd)) | 8.58 (4.25) | 0 | 20 |
| Smoking (%) |  |  |  |
| No | 60 (74.1) |  |  |
| Not currently | 7 (8.6) |  |  |
| Yes | 14 (17.3) |  |  |
| Cigarettes (%) |  |  |  |
|  | 68 (82.9) |  |  |
| 0 | 3 (3.7) |  |  |
| 1/2 PACK | 1 (1.2) |  |  |
| 10 CIG | 1 (1.2) |  |  |
| 15 CIGS | 1 (1.2) |  |  |
| 2 PACKS | 1 (1.2) |  |  |
| 2-3 CIGS | 1 (1.2) |  |  |
| 5 CIGS | 2 (2.4) |  |  |
| 5-10 | 1 (1.2) |  |  |
| 6 CIGS | 2 (2.4) |  |  |
| 6-10 CIG | 1 (1.2) |  |  |
| Smoking during pregnancy = Yes (%) | 10 (12.8) |  |  |
| Cigarettes during pregnancy (%) | | |  |
|  | 71 (86.6) |  |  |
| 0 | 2 (2.4) |  |  |
| 1 CIG | 2 (2.4) |  |  |
| 1/2 PACK | 1 (1.2) |  |  |
| 2 CIGS | 1 (1.2) |  |  |
| 2 PACKS | 1 (1.2) |  |  |
| 2-3 CIGS | 1 (1.2) |  |  |
| 2-4 CIGS | 1 (1.2) |  |  |
| 4-6 CIGS | 1 (1.2) |  |  |
| 5 CIGS | 1 (1.2) |  |  |
| Drinking during pregnancy = Yes (%) | 1 (1.2) |  |  |
| Maternal drug use = Yes (%) | 2 (2.5) |  |  |
| Maternal drugs during pregnancy = Yes (%) | 4 (5.0) |  |  |
| Steroids = Yes (%) | 53 (67.9) |  |  |
| Maternal antibiotic = Yes (%) | 36 (48.0) |  |  |
| Delivery method = Vaginal (%) | 18 (22.2) |  |  |
| Labor length (hrs) (%) | |  |  |
|  | 48 (58.5) |  |  |
| <1 hr | 7 (8.5) |  |  |
| 1-10 hrs | 18 (22.0) |  |  |
| 11-20 hrs | 4 (4.9) |  |  |
| 21-30 hrs | 2 (2.4) |  |  |
| 41-50 hrs | 1 (1.2) |  |  |
| 51-60 hrs | 1 (1.2) |  |  |
| 71-80 hrs | 1 (1.2) |  |  |
| Infant gender = Male (%) | 40 (48.8) |  |  |
| Maternal weight at delivery (lbs) (mean (sd)) | 194.17 (49.93) | 102 | 313 |
| Anesthesia (%) |  |  |  |
| 0 | 7 (9.5) |  |  |
| Epidural | 10 (13.5) |  |  |
| General | 29 (39.2) |  |  |
| None | 2 (2.7) |  |  |
| Spinal | 26 (35.1) |  |  |
| Delivery complications = Yes (%) | 18 (23.7) |  |  |
| Infant weight at delivery (g) (mean (sd)) | 1086.71 (218.49) | 600 | 1485 |
| APGAR 1 min (mean (sd)) | 6.09 (1.98) | 1 | 9 |
| APGAR 5MIN (mean (sd)) | 7.53 (1.51) | 3 | 9 |
| SNAPPEII FINAL (mean (sd)) | 18.99 (16.51) | 0 | 81 |
| SNAPII FINAL (mean (sd)) | 10.08 (9.51) | 0 | 54 |
| SNAPII week1 (mean (sd)) | 11.73 (11.70) | 0 | 66 |
| SNAPII week2 (mean (sd)) | 3.53 (6.19) | 0 | 32 |
| SNAPII week3 (mean (sd)) | 2.47 (5.83) | 0 | 35 |
| SNAPII week4 (mean (sd)) | 0.79 (3.72) | 0 | 24 |
| SNAPII week5 (mean (sd)) | 0.53 (2.34) | 0 | 14 |
| SNAPII week6 (mean (sd)) | 0.84 (3.42) | 0 | 19 |
| Gestational age at delivery (weeks) (mean (sd)) | 28.44 (2.39) | 24 | 37 |
| Time to enteral feed (days) (mean (sd)) | 12.25 (5.06) | 5 | 39 |
| Infant weight at 6WKS (g) (mean (sd)) | 1887.91 (335.23) | 1210 | 2700 |
| Infant weight at discharge (g) (mean (sd)) | 2678.96 (900.13) | 1101 | 6500 |
| ROPn (%) |  |  |  |
| No | 67 (84.8) |  |  |
| Stg1 | 9 (11.4) |  |  |
| Stg2 | 3 (3.8) |  |  |
| BPDn = Yes (%) | 5 (6.3) |  |  |
| SEPSIS = Yes (%) | 10 (12.7) |  |  |
| NEC = Yes (%) | 3 (3.8) |  |  |
| IVHn (%) |  |  |  |
| No | 66 (88.0) |  |  |
| Stg1 | 4 (5.3) |  |  |
| Stg2 | 1 (1.3) |  |  |
| Stg3 | 1 (1.3) |  |  |
| Stg4 | 3 (4.0) |  |  |
| TRANSFUSIONS = Yes (%) | 34 (43.0) |  |  |
| Days on Oxygen (mean (sd)) | 14.29 (20.82) | 0 | 101 |
| Length of Stay (days) (mean (sd)) | 69.84 (36.74) | 20 | 215 |
| Feed Intolerance = Yes (%) | 15 (19.5) |  |  |
| Multiples (%) |  |  |  |
| singleton | 54 (74.0) |  |  |
| triplet | 3 (4.1) |  |  |
| twin | 16 (21.9) |  |  |
| Born at TGH = Yes (%) | 75 (94.9) |  |  |
| Deceased = Yes (%) | 1 (1.4) |  |  |
| Maternal BMI (mean (sd)) | 28.08 (7.45) | |  |
