# Supplementary figures and images for: Longitudinal Microbiome Composition and Stability Correlate with Increased Weight and Length of Very-Low-Birth-Weight Infants
Source: mSystems. 2019 Feb 26;4(1):e00229-18. doi: 10.1128/mSystems.00229-18 (PMC6392092; doi:10.1128/mSystems.00229-18)

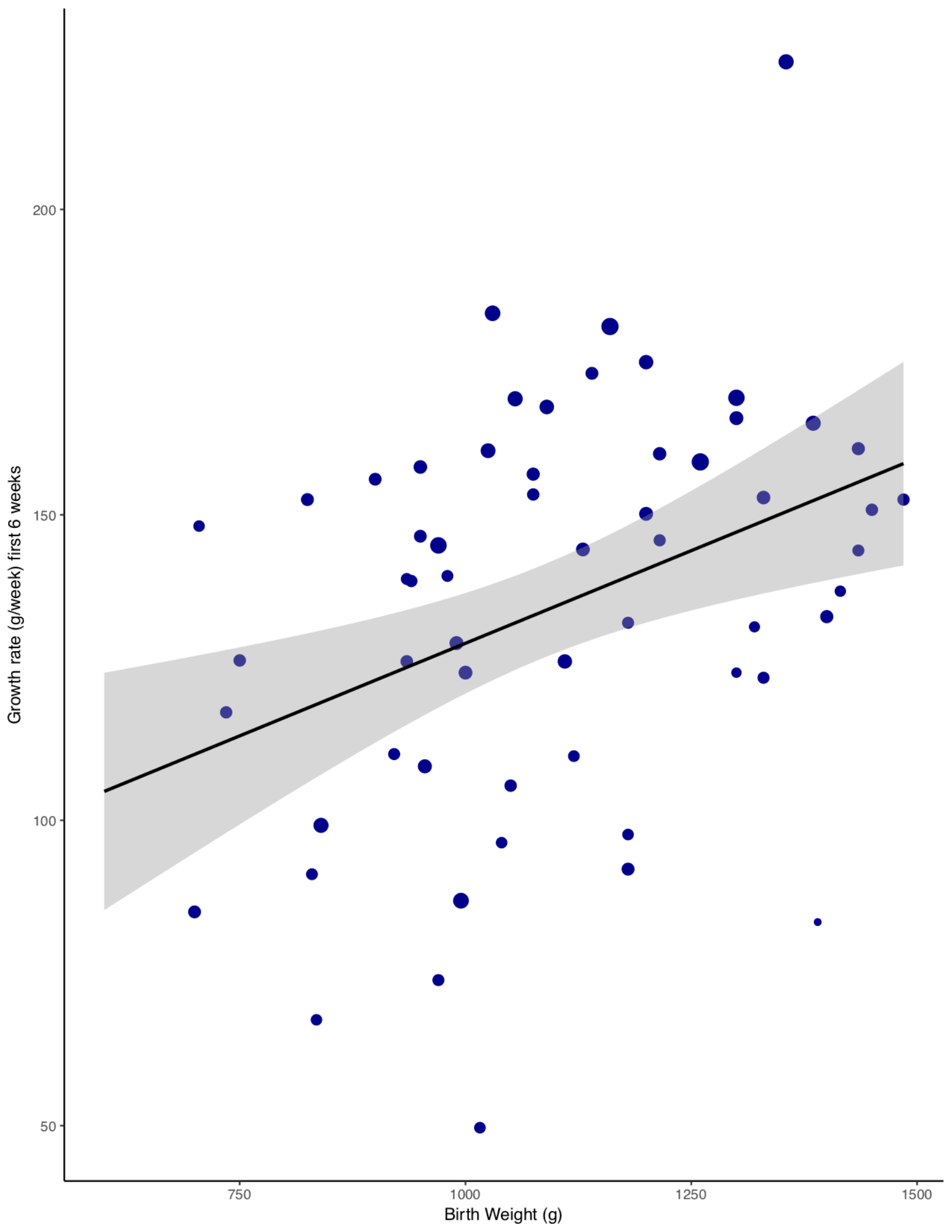

Supplement: FIG S1 [file mSystems.00229-18-sf001.pdf]

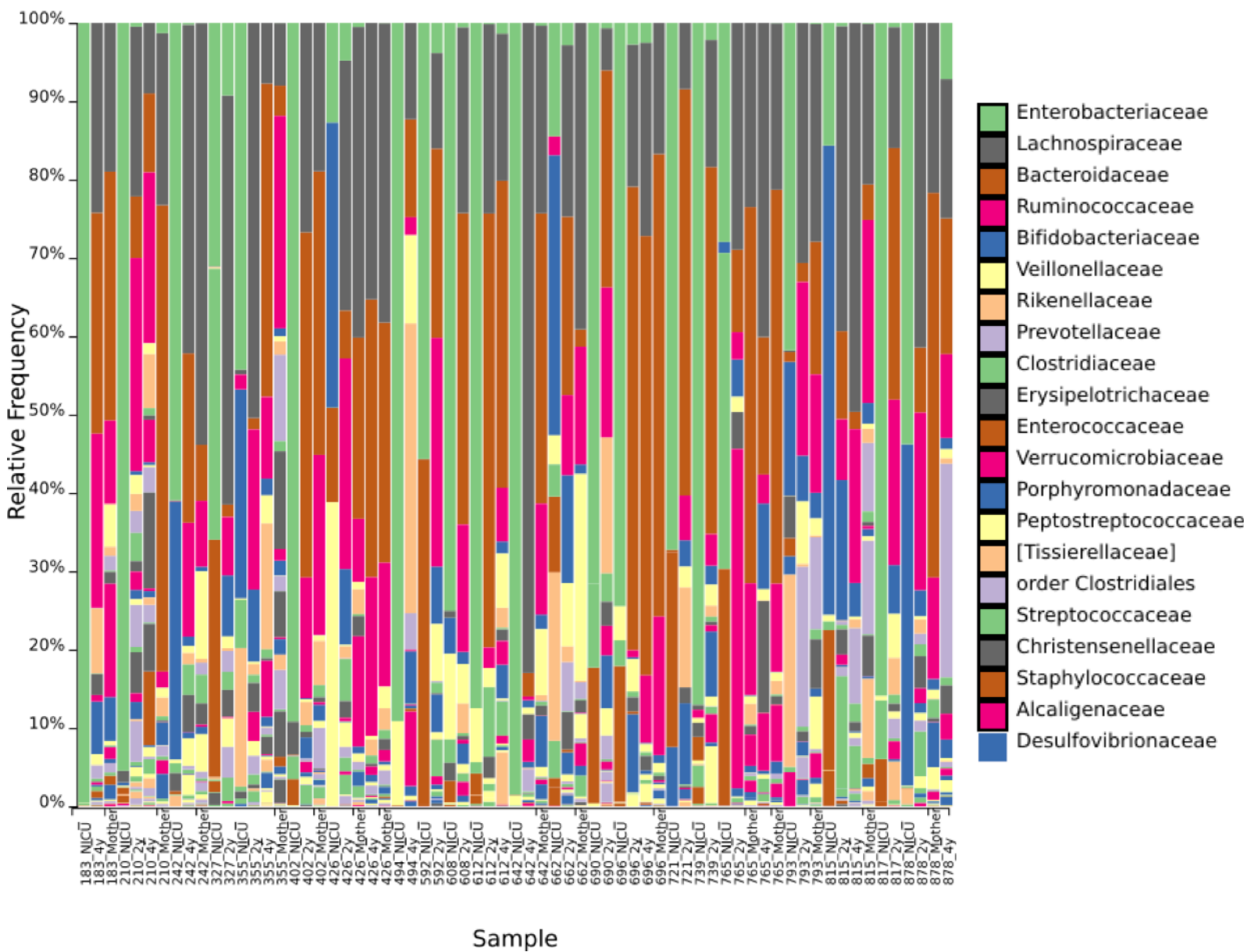

Supplement: FIG S2 [file mSystems.00229-18-sf002.pdf]
