# Supplementary material for: Longitudinal Microbiome Composition and Stability Correlate with Increased Weight and Length of Very-Low-Birth-Weight Infants
Source: mSystems. 2019 Feb 26;4(1):e00229-18. doi: 10.1128/mSystems.00229-18 (PMC6392092; doi:10.1128/mSystems.00229-18)
Supplement: TABLE S2 [file mSystems.00229-18-st002.docx]

| taxonomy |
| --- |
| k__Bacteria; p__Firmicutes; c__Clostridia; o__Clostridiales; f__Lachnospiraceae; g__[Ruminococcus]; s__gnavus |
| k__Bacteria; p__Proteobacteria; c__Gammaproteobacteria; o__Pasteurellales; f__Pasteurellaceae; g__Haemophilus; s__parainfluenzae |
| k__Bacteria; p__Firmicutes; c__Clostridia; o__Clostridiales; f__Ruminococcaceae; g__Oscillospira; s__ |
| k__Bacteria; p__Bacteroidetes; c__Bacteroidia; o__Bacteroidales; f__Bacteroidaceae; g__Bacteroides; s__ |
| k__Bacteria; p__Firmicutes; c__Bacilli; o__Lactobacillales; f__Streptococcaceae; g__Streptococcus; s__ |
| k__Bacteria; p__Firmicutes; c__Clostridia; o__Clostridiales; f__Peptostreptococcaceae; g__; s__ |
| k__Bacteria; p__Firmicutes; c__Clostridia; o__Clostridiales; f__Lachnospiraceae; g__[Ruminococcus]; s__torques |
| k__Bacteria; p__Actinobacteria; c__Actinobacteria; o__Bifidobacteriales; f__Bifidobacteriaceae; g__Bifidobacterium |
| k__Bacteria; p__Proteobacteria; c__Gammaproteobacteria; o__Enterobacteriales; f__Enterobacteriaceae; g__Escherichia; s__coli |
| k__Bacteria; p__Actinobacteria; c__Coriobacteriia; o__Coriobacteriales; f__Coriobacteriaceae; g__Eggerthella; s__lenta |
| k__Bacteria; p__Bacteroidetes; c__Bacteroidia; o__Bacteroidales; f__Rikenellaceae; g__; s__ |
| k__Bacteria; p__Firmicutes; c__Clostridia; o__Clostridiales; f__Lachnospiraceae; g__Dorea; s__ |
| k__Bacteria; p__Firmicutes; c__Bacilli; o__Lactobacillales; f__Streptococcaceae; g__Streptococcus; s__ |
| k__Bacteria; p__Firmicutes; c__Clostridia; o__Clostridiales; f__Lachnospiraceae; g__; s__ |
